# Complete Genome Sequence of a Dickeya fangzhongdai Type Strain Causing Bleeding Canker of Pear Tree Trunks

**DOI:** 10.1128/genomeA.00177-18

**Published:** 2018-05-17

**Authors:** Yuqiang Zhao, Yanli Tian, Xiang Li, Baishi Hu

**Affiliations:** a Institute of Botany, Jiangsu Province and Chinese Academy of Sciences, Nanjing Botanical Garden Mem. Sun Yat-Sen, Jiangsu Key Laboratory for the Research and Utilization of Plant Resources, Nanjing, China; b College of Plant Protection and Key Laboratory of Integrated Management of Crop Diseases and Pests, Ministry of Education, Nanjing Agricultural University, Nanjing, China; c Charlottetown Laboratory, Canadian Food Inspection Agency, Charlottetown, Prince Edward Island, Canada

## Abstract

Dickeya fangzhongdai DSM 101947^T^ was isolated from pear trees (Pyrus pyrifolia) in Zhejiang Province, China, and is the causal agent of bleeding canker, a devastating disease of pear trees. Here, we provide the complete genome sequence of this bacterium, which has a 5,027,163-bp-long genome, including 4,375 protein-coding genes and 100 RNA genes. This strain possesses a number of genes encoding virulence factors of pathogens.

## GENOME ANNOUNCEMENT

Bleeding canker of tree trunks is a devastating disease of pear trees in China (1). Dickeya fangzhongdai is the causal agent of bleeding canker, a pathogen that uniquely affects wood plants in seven species of Dickeya (2). For studies on the pathogenic mechanism, we report here the *de novo*-sequenced complete genome of type strain DSM 101947 from a pear trunk displaying bleeding canker symptoms in Zhejiang Province, China, in August 2009.

The genomic DNA was isolated using the QIAamp DNA minikit (Qiagen, Germantown, MD), according to the manufacturer’s instructions. DNA quality was checked using a NanoDrop spectrophotometer (Thermo Scientific) and quantitated using a Qubit 2.0 fluorometer (Life Technologies) and Agilent 2100 Bioanalyzer (Agilent Technologies, Santa Clara, CA). The whole-genome sequence of strain DSM 101947^T^ was sequenced using paired-end Illumina HiSeq sequencing technology with TruSeq version 3 chemistry (Majorbio). This resulted in 148 contigs, with an *N*_50_ value of 111,372 bp, *N*_90_ value of 36,275 bp, and genome size of 5,027,163 bp at 150× coverage. The assembled DSM 101947^T^ genome revealed a single circular chromosome and an overall G+C content of 56.76%. A total of 4,375 protein-coding genes were predicted in the genome, and 78 tRNAs and 22 rRNAs were found in the chromosome. In addition, DSM 101947^T^ has 1 small plasmid of 5,284 bp containing 5 protein-coding genes.

### Accession number(s).

The complete genome sequence of Dickeya fangzhongdai DSM 101947^T^ has been deposited in GenBank under the accession numbers CP025003 (chromosome) and CP025004 (plasmid).
